# Zunyimycins B and C, New Chloroanthrabenzoxocinones Antibiotics against Methicillin-Resistant *Staphylococcus aureus* and *Enterococci* from *Streptomyces* sp. FJS31-2

**DOI:** 10.3390/molecules22020251

**Published:** 2017-02-08

**Authors:** Yuhong Lü, Meiyun Shao, Yinyin Wang, Shengyan Qian, Miao Wang, Yingquan Wang, Xiaoqian Li, Yuxin Bao, Chengmin Deng, Changwu Yue, Daishun Liu, Ning Liu, Minghao Liu, Ying Huang, Zehui Chen, Yonglin Hu

**Affiliations:** 1 Guizhou Key Laboratory of Microbial Resources & Drug Development, Zunyi Medical University, Zunyi 563003, Guizhou, China; 18585737637@163.com (Y.L.); meiyun216@126.com (M.S.); yinyinwang0530@126.com (Y.W.); qianshengyan@126.com (S.Q.); wangmiao_good@163.com (M.W.); yingquanwang@126.com (Y.W.); xiaoqianli0908@163.com (X.L.); baoyuxin8680915@126.com (Y.B.); 2 Zunyi Key Laboratory of Genetic Diagnosis & Targeted Drug Therapy, The First People’s Hospital of Zunyi, Zunyi 563003, Guizhou, China; dengcmin@126.com; 3 State Key Laboratory of Microbial Resources, Institute of Microbiology, Chinese Academy of Sciences, Beijing 100101, China; fussliu@126.com (N.L.); lysf1987313@163.com (M.L.); 4 Department of Clinical Laboratory Medicine, Zunyi Medical University, Zunyi 563003, Guizhou, China; czhtyb@163.com (Z.C.); huyonglin5559@163.com (Y.H.)

**Keywords:** zunyimycins, chloroanthrabenzoxocinones, antibacterial, activity, MRSA, *Enterococci*, streptomycetes

## Abstract

This study performed an optimization of the fermentation conditions to activate the expression of the zunyimycin family biosynthesis genes of the zunyimycin-producing streptomycetes strain *Streptomyces* sp. FJS31-2. Bioassay-guided isolation and purification by varied chromatographic methods yielded two new compounds of the zunyimycin derivatives, namely, 31-2-7 and 31-2-8, accompanied with three known anthrabenzoxocinones family members of zunyimycin A, BE24566B, and chloroanthrabenzoxocinone. Their structures were elucidated by NMR, HRESIMS, IR, UV, and CD. Results showed that these two compounds were structurally similar to the previously reported compound zunyimycin A but differed in positions and number of chlorine atom substitution. The two novel compounds were called zunyimycins B and C. Antibacterial activity assay indicated that zunyimycin C showed a good inhibitory effect on the methicillin-resistant *Staphylococcus aureus* and *Enterococci*.

## 1. Introduction

**Figure 1:**
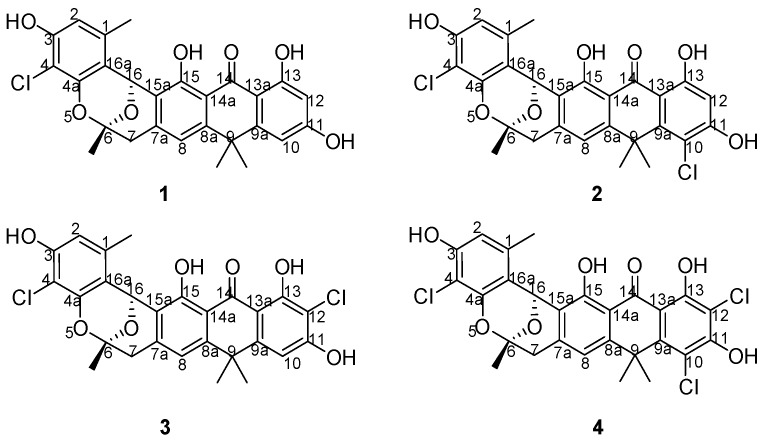
Chemical structure of zunyimycins from *Streptomyces* sp. FJS31-2.

Actinomycetes were known as the most important microorganisms for microbial-derived halogenated antibiotics because they produce numerous novel halogenated natural products with a variety of biological activities [1,2]. Currently, hundreds of actinomycete-derived halogenide with potent pharmacological activities have been reported [3], including the well-known nonribosomal peptide, vancomycin, which is extensively used to treat infections caused by methicillin-resistant *Staphylococcus aureus* [4,5]. However, antibiotics, including vancomycin, are also powerless when faced with the superbugs [6,7]. Accordingly, researchers have been desperately seeking new, effective drug options because of the potential for “no prescription” to occur [8]. Genome mining is an effective tool that is employed by an increasing number of scientists for novel natural product discovery from actinomycetes [9,10]. During the course of our investigation for novel halogenated natural products from streptomycetes [11], we isolated the chlorinated modified compounds of the type II polyketide BE24566B (**1**) and its chloroderivatives called zunyimycin A (**2**) from *Streptomyces* sp. FJS31-2 [12,13]. The current study reports the isolation and characterization of two new halogenated type II polyketides zunyimycin B (**3**) and zunyimycin C (**4**) accompanied by their three derivatives of zunyimycin A, BE24566B, and chloroanthrabenzoxocinone from the fermentation solid culture of *Streptomyces* sp. FJS31-2 (Figure 1). Antibacterial activity assay indicated that zunyimycins B and C showed good inhibitory effects on the methicillin-resistant *S. aureus* and *Enterococci*.

## 2. Results

### 2.1. Biosynthesis of Zunyimycins

**Figure 2:**
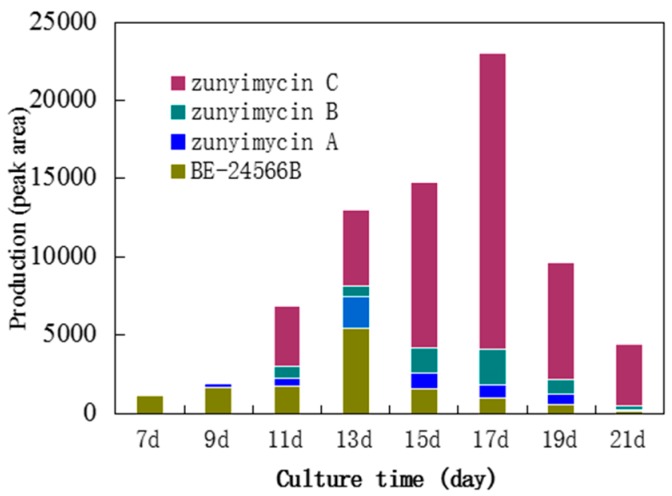
Biosynthesis model of the zunyimycin family.

Among the 23 fermentation media, which were designed to screen the most effective medium for the biosynthesis of zunyimycins under the same conditions, Medium 22 was selected as the fermentation medium for zunyimycins because it exhibited the highest produced efficiency both on the product variety and the output among the four zunyimycin family numbers detected by HRESI-MS (Table 1). The results of the production testing of compounds by HPLC of the crude extracts from *Streptomyces* sp. FJS31-2 suggested that the four numbers of the zunyimycin family showed a different producing model under different incubation time (Figure 2). For example, the initiation of the biosynthesis of BE-24566B may be earlier than seven days and reach its peak on the 13th day. By contrast, zunyimycin A may start on the 9th day and reach its peak on the 13th day as well.

### 2.2. Chemical Identification of Zunyimycin B and C

Zunyimycin B (**3**) was obtained a pale yellow powder and is soluble in dimethyl sulfoxide, MeOH, and MeCOMe, among others. The IR spectrum of **1** showed absorption at 3385 cm^−1^ and 1609 cm^−1^ indicative of the presence of hydroxyl and carbonyl groups and the absorption at 1421 cm^−1^ and 1384 cm^−1^ illustrated the existence of benzene ring. HR-TOF-MS ion of the IR spectrum of **1** at *m*/*z* 527.0658 [M]^−^ indicated the molecular formula to be C_27_H_22_Cl_2_O_7_, thereby implying 16 degrees of unsaturation. The ^13^C-NMR, HSQC, and DEPT spectra of **1** displayed signals for 18 aromatic carbons at δ_C_ 160.6, 157.7, 152.6, 151.8, 150.7, 150.7, 150.4, 141.2, 133.4, 122.5, 117.2, 115.7, 113.6, 111.2, 107.2, 106.3, and 101.7 which was similar to the ^13^C-NMR, HSQC, and DEPT spectra of BE-24566B (δ_C_ 167.7, 167.4, 159.6, 159.0, 157.0, 154.5, 152.4, 144.8, 138.0, 125.5, 119.5, 115.8, 113.2, 109.1, 108.4, 103.0, and 101.2) and zunyimycin A (δ_C_ 164.5, 163.0, 156.9, 153.2, 152.6, 150.6, 148.3, 141.9, 133.4, 122.1, 117.7, 115.4, 112.6, 109.9, 107.6, 102.6, and 101.6) demonstrated that the constituent was anthrabenzoxocinone (ABX). In addition, combined with the signal of carbonyl group of δ_C_ 189.7, the methyl group at δ_C_ 39.4, quaternary carbon at δ_C_ 38.1, and carbon proton at δ_C_ 98.4 indicated that Compound **3** had the skeleton of ABX. Moreover, the ^1^H-NMR spectra of **1** that display signals at 6.82 (1H, s), 6.57 (1H, s), 6.17 (1H, s), 2.41 (3H, s), 1.51 (3H, s), 1.45 (3H, s), and 1.36 (3H, s) also demonstrate the presence of ABX. Table 2 shows the NMR data. The HMBC experiment showed correlation between the proton at δ_H_ 6.82 and carbons at δ_C_ 111.2 and 122.5, thereby indicating that one hydroxyl atom connected with the C-8 position. The signal at δ_H_ 6.57 (1H, s) and carbons at δ_C_ 106.3, combined with the ROSEY spectra showed a correlation with H-10/9-CH_3_, thereby implying that the signal at δ_H_ 6.57 connected with the benzene ring at the C-10 position. The proton signal at δ_H_ 6.17 (1H, s) correlated with δ_C_ 152.6, 150.7, and 133.4, thereby indicating that the signals to δ_H_ 6.17 connected with the benzene ring at the C-2 position. The ^1^H–^1^H COSY showed that the correlation H-2/1-CH_3_ can also demonstrate the δ_H_ 6.17 connected with the C-2 position. In accordance with the preceding information, the relative structure of Compound **3** was substituted by chlorine atoms at the C-4 and C-12 positions of ABX.

**Figure 3:**
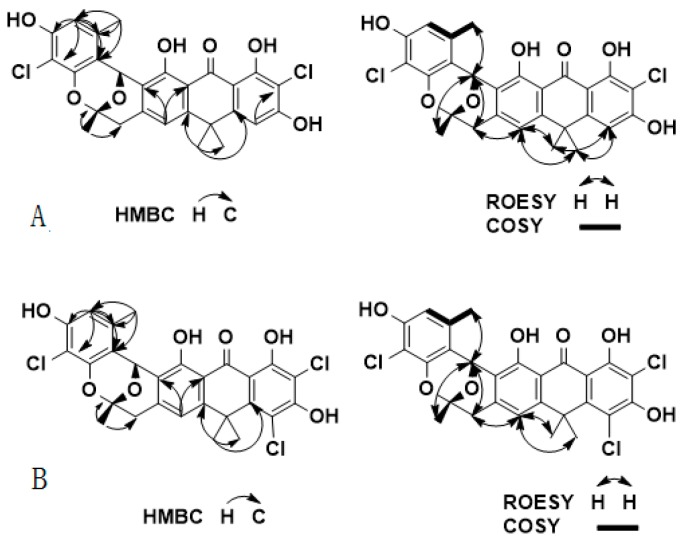
HMBC and ROESY correlations for Zunyimycin B (upper, **A**) and C (lower, **B**).

Zunyimycin C (**4**) was obtained as a pale yellow powder. Its molecular formula C_27_H_21_Cl_3_O_7_ established on the basis of the HR-TOF-MS ion at *m*/*z* 527.0658 [M]^−^ implied that it is one chlorine atom more than zunyimycin B. The analysis of ^1^H-, ^13^C-NMR, DEPT, COSY, and HSQC spectra (Table 2) was similar with zunyimycin B, thereby confirming that zunyimycin C had the presence of ABX. The HMBC experiment showed a correlation between the proton at δ_H_ 6.86 and carbons at δ_C_ 112.3 and 122.1, thereby indicating that one hydroxyl atom is connected with the C-8 position. The proton signal at δ_H_ 6.17 (1H, s) correlated with δ_C_ 152.6, 150.7, 133.4, 115.7, and 113.6, thereby indicating that the signals to δ_H_ 6.17 is connected with the benzene ring at the C-2 position. The ^1^H-^1^H COSY spectra showed that the correlation H-2/1-CH3 can also demonstrate the δ_H_ 6.17 connected with the C-2 position. In accordance with the preceding information, the relative structure of Compound **2** was substituted by chlorine atoms at the C-4, C-10, and C-12 positions of ABX. Figure 3 shows the 2D NMR data.

**Figure 4:**
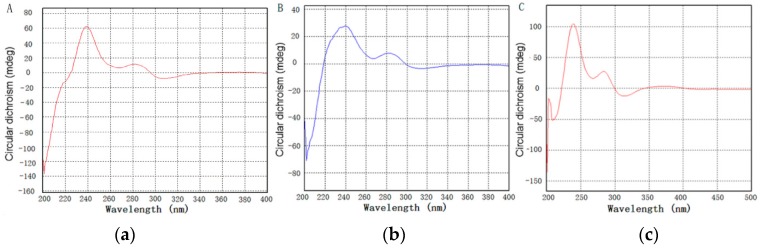
Circular dichroism spectrum of zunyimycin A (**a**); zunyimycin B (**b**) and zunyimycin C (**c**).

The absolute configuration of zunyimycins B and C was confirmed through the CD spectrum (Figure 4). The CD spectrum of zunyimycins B and C displayed an apparent positive cotton effect (CD) at 239 nm and at 283 nm, thereby indicating 2 S configurations for the C-16 and C-6 positions in the two compounds.

### 2.3. Antibacterial Activity of Zunyimycins

The results of the antibacterial activity testing showed that both zunyimycins A, B and C exhibited antibacterial activity against the *Enterococci (Enterococcus faecalis), Bacillus* (*Bacillus subtilis*) as well as both the methicillin-sensitive and methicillin-resistant *Staphylococcus aureus*. Among the three chloroanthrabenzoxocinones antibiotics, zunyimycin C showing more higher antibacterial activity since the MICs against the *Enterococci*, *Bacillus* as well as both the methicillin-sensitive and methicillin-resistant *Staphylococcus aureus* were lower than its analogues of zunyimycin A and B (Table 3).

## 3. Discussion

**Figure 5:**
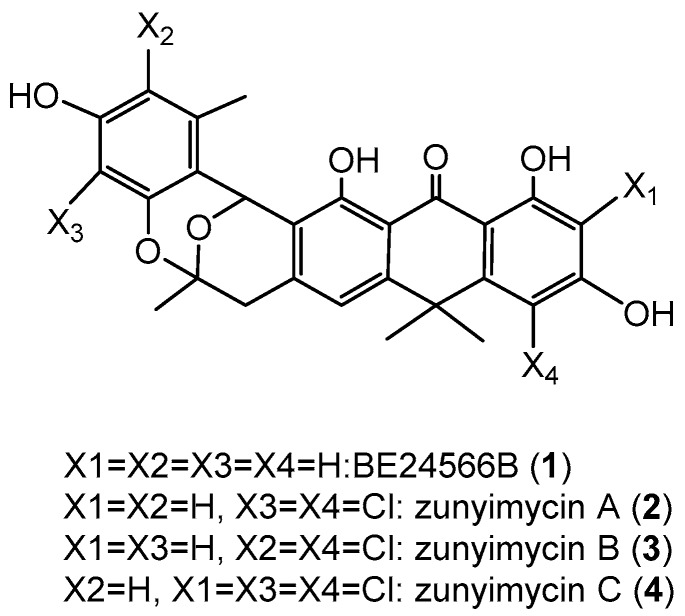
Proposed catalytic sites of AbxH.

Currently, over 4500 halogenated natural products were isolated and generally with various biological activities, such as antibacterial, antitumor, and antiviral activities [14]. Halogenated natural products have been known as the most important resource of antibiotics because of their biological activity and their potential for medicinal use [15,16]. ABXs are a group of hexacyclics aromatic ketones with bioactivities [17], which were first isolated from *Streptomyces violaceusniger*. In the current study, two novel compounds, namely zunyimycins B and C, showed good inhibitory effect on the methicillin-resistant *S. aureus*, which were isolated from *Streptomyces* sp. FJS31-2 along with their analogues zunyimycin A and BE-24566B. In accordance with the results of the structure elucidation of zunyimycins A, B, and C, four modification sites for halogenation were discovered (Figure 5). The halogenation reactions of the three compounds may be conducted by AbxH because only one halogenase (AbxH) was scanned in the genome of *Streptomyces* sp. FJS31-2. In spite of the catalytic mechanism that has not been precisely defined, AbxH may play an important role in the discovery of actinomycete-derived halogenated natural products. The halometabolites are not only highly diverse in biogenic origin, but also with respect to the halogen substitution patterns as well as chemical and structural complexity. Most halometabolites are biologically active, showing, e.g., antimicrobial, antifungal, or antibiotic activity. Since the biological activities of halometabolites are usually critically dependent on the presence (halogenated modification sites or number) of the halogen(s) [18], this may the reason of zunyimycin C shows the best activities compared to its analogues.

The results of the biosynthesis model analysis of the zunyimycin family suggest that under the same conditions, fermentation time may affect the output and compounds during fermentation. Hence, unlike BE-24566B and zunyimycin A, the biosynthesis of zunyimycins B and C started on the d11th day and reached its peak on the 17th day caused by the chloro-halogenation reaction of the compounds happening after the formation of the backbone of the compounds (Figure 2).

## 4. Materials and Methods

### 4.1. Strains and Medium

Five methicillin resistant *Staphylococcus* *aureus* clinical isolates and four *Enterococcus faecalis* clinical isolates were obtained from the Department of Laboratory Medicine of Zunyi Medical University. *Staphylocccus aureus* (ATCC: 29213), *Enterococcus faecalis* (ATCC: 29212) and *Bacillus subtilis* (CGMCC: 1.2428) were from China General Microbiological Culture Collection Center. *Streptomyces* sp. FJS31-2 was isolated from a soil sample collected from the Fanjing Mountain of Guizhou Province and was deposited in the China General Microbiological Culture Collection Center under accession number CGMCC 4.7321. The actinomycetic strain was preliminarily identified as a *Streptomyces* species based on morphological observation and physio-biochemical characteristics. The 16S rRNA gene was cloned by PCR and the DNA sequencing showing that it was substantially homologous (i.e., above 99%) with the *Streptomyces sparsogenes* strain NBRC 13086 by multiple sequence alignment and phylogeny evolution analysis revealed that it was a variant species of *S. sparsogenes*. The genome DNA of *Streptomyces* sp. FJS31-2 was sequenced and deposited in GenBank under accession number PRJNA320463. The results of the antibacterial activity assay suggest that the strain exhibits biological activity against *Candida albicans*, *B. subtilis*, and *M. luteus*. The gene screening results of the secondary metabolism biosynthesis-associated genes showed that the coding region DNA sequence of halogenase, non-ribosomal peptide synthetases, type I polyketide synthase, and type II polyketide synthase (PKS II) were detected in the genome of *Streptomyces* sp. FJS31-2.

A total of 23 media with different carbon sources, nitrogen sources, and humic acid extract were designed to induce the biosynthesis of zunyimycins (Table 4). After culturing under the same conditions (28 °C, stationary culture) for 7, 9, 11, 13, 15, 17, 19, and 21 days, the lawn plate including streptomycetes and culture medium were extracted with the same volume of ethyl acetate thrice. The production of the target compound from different media was detected through HPLC in accordance with the peak area of the ultraviolet absorption spectrum to optimize the high-producing culture condition of *Streptomyces* sp. FJS31-2 for zunyimycins.

### 4.2. Fermentation, Isolation, and Chemical Identification of BE-24566B and Zunyimycins A, B, and C

In accordance with the results of the optimized culture conditions, *Streptomyces* sp. FJS31-2 was cultured using 140 × 500 mL shake flasks containing 100 mL of ISP 2 agar medium with 10% natural humus acid water extracts. Thereafter, the culture was incubated for 13 days at 28 °C to produce zunyimycin B and 17 days for zunyimycin C. The solid culture was mashed and extracted thrice with 100 mL of ethanol in a shake flask at 28 °C for 7 h at 110 rpm after cultivation. Thereafter, the organic portion was concentrated in vacuo to remove the solvent. The crude extract was applied to silica gel column chromatography using the CHCl_3_/MeOH gradient to obtain the crude products. Further purification was conducted using Sephadex LH-20 (GE Healthcare, Tokyo, Japan) (MeOH) column and RP-HPLC (Shimadzu SPD-M20A with Xbridge ODS 10 mm × 150 mm column). Compounds were identified using a HRESI-MS (Waters Xevo G2 QTOF mass spectrometer (Waters Corporation, Milford, MA, USA) and NMR (Bruker AV 600 MHz) (Bruker Corporation, Karlsruhe, Germany) for analysis [19].

### 4.3. Antibacterial Activity Assay

The antibacterial activity of zunyimycins A, B, and C was investigated against *Staphylococcus aureus* and *Enterococci*. Bacteria were grown at 37 °C in Luria-Bertani (LB) broth (Difco, Sparks, MD, USA) with continuous shaking until the optical density (OD_595_) reached 0.6. The bactericidal activity of zunyimycins A, B, and C was investigated using parameters such as the minimum inhibitory concentrations (MIC). The MIC values for zunyimycins A, B, and C were evaluated in quadruplet wells of sterile 96-well microtiter plates using the broth microdilution assay using the previously described broth microdilution procedure. Briefly, bacterial strains were clutured overnight at 35 °C in liquid LB medium and test strains were suspended in fresh LB to yield a final density of 5 × 10^5^ clolonies-forming units (cfu)/mL [20]. Geometric dilution ranging from 100 to 0.05 µg of zunyimycins and DMSO solvent were prepared in a 96-well microtiter plate (50 µL of LB, 50 µL of the zunyimycins and pure DMSO +50 µL of test strains). All plates were sealed lightly (with ventilation) and incubated thereafter at 35 °C for 18 h. The bacterial growth was indicated by the presence of white “pellet” on the well bottom. All tests were performed in quadruplets for test strains. The concentration of the first well with no turbidity was considered the MIC.

## 5. Conclusions

In summary, two novel compounds, namely zunyimycins B and C along with their analogous zunyimycin A and BE-24566B, were isolated from *Streptomyces* sp. FJS31-2. An antibacterial activity assay indicated that zunyimycins A, B and C showed antibiotic activity against methicillin-resistant *S. aureus*.

## Figures and Tables

**Table 1:** Biosynthesis of zunyimycins.

Medium	Production (Peak Height, mAU)			
	BE24566B	Zunyimycin A	Zunyimycin B	Zunyimycin C
1				
2	107			
3				
4				
5	50			
6	320		135	190
7				
8	124			
9	122			
10	200	52	24	
11				90
12	75			
13				
14	150			
15	115			
16	110			
17				
18			100	108
19	99			
20	160			
21				
*22*	*525*	*250*	*526*	*824*
23	117			150

**Table 2:** NMR data of zunyimycin B and C in 500 (^1^H) and 125 (^13^C) MHz (MeOH-*d*_6_, δppm).

Position	^1^H		^13^C	
	Zunyimycin B	Zunyimycin C	Zunyimycin B	Zunyimycin C
1			133.4	133.4
2	6.17 (1H, s)	6.27 (1H, s)	101.7	101.7
3			150.7	150.7
4			113.6	113.6
4a			152.6	152.6
5				
6			98.4	98.3
7	3.04 (1H, d, *J* = 11.3) 2.93 (1H, d, *J* = 17.5)	3.04–3.11 (2H, m)	39.6	39.7
7a			141.2	142.4
8	6.82 (1H, s)	6.86 (1H, s)	117.2	117.9
8a			150.7	146.4
9			38.1	39.4
9a			150.4	153.0
10	6.57 (1H, s)		107.2	107.9
11			160.6	159.6
12			106.3	107.8
13			151.8	157.4
13a			113.6	109.7
14			189.7	190.8
14a			111.2	112.3
15			157.7	157.1
15a			122.5	122.1
16	6.10 (1H, s)	6.10 (1H, s)	65.8	65.7
16a			115.7	115.6
1-CH_3_	2.52 (3H, s)	2.49 (3H, s)	15.6	15.8
6-CH_3_	1.51 (3H, s)	1.60 (3H, s)	26.2	26.2
9-CH_3_	1.36 (3H, s)	1.79 (3H, s)	33.0	27.5
9-CH_3_	1.45 (3H, s)	1.69 (3H, s)	32.6	27.8

**Table 3:** Minimum inhibitory concentrations of zunyimycins (µg/mL).

Strains	Ampicillin	Zunyimycin A	Zunyimycin B	Zunyimycin C
*S. aureus* (ATCC: 29213)	1.05	3.44	3.94	0.94
MRSA clinical isolates (08301)	>100	6.89	7.88	3.75
MRSA clinical isolates (161222330)	>100	16.71	25.62	8.14
MRSA clinical isolates (161231380)	>100	8.36	12.81	4.07
MRSA clinical isolates (170108317)	>100	16.71	25.62	4.07
MRSA clinical isolates (161231350)	>100	16.71	25.62	4.07
*E. faecalis* (ATCC: 29212)	>100	13.78	15.75	7.50
*E. faecalis* clinical isolates (160803348)	>100	16.71	12.81	4.07
*E. faecalis* clinical isolates (160804314)	>100	16.71	12.81	8.14
*E. faecalis* clinical isolates (161222328)	>100	33.43	12.81	4.07
*E. faecalis* clinical isolates (170106034)	>100	16.71	25.62	8.14
*B. subtilis* (CGMCC: 1.2428)	0.71	13.78	15.75	3.75

**Table 4:** Medium for the biosynthesis of zunyimycins.

Medium Components (g/L)								
Medium	CaCO_3_	Glucose	Malt Exact	Yeast Exact	Mannitol	NH_4_NO_3_	Humic Acid A	Humic Acid B
1	2		10		4	4		
2	2		10	4	4			
3	2		10	1	4			
4	2		10	2	4	2		
5	2	1	10			4		
6	2	1	10	4				
7	2	1	10	1				
8	2	1	10	2		2		
9	2	4	10			4		
10	2	4	10	4				
11	2	4	10	1				
12	2	4	10	2		2		
13	2	2	10		2	4		
14	2	2	10	4	2			
15	2	2	10	1	2			
16	2	2	10	2	2	2		
17	2	1		1				
18	2	4	10	4			0.5	
19	2	4	10	4			1.	
20	2	4	10	4			1.5	
21	2	4	10	4				0.5
*22*	*2*	*4*	*10*	*4*				*1.0*
23	2	4	10	4				1.5

Humic acid A: extract with water; Humic acid B: extract with alcohol. Each medium was supplemented with 1 mL trace elements (ZnSO_47_·H_2_O 1 g/L, FeSO_47_·H_2_O 1 g/L, MnCl_2_·4H_2_O 1 g/L,·CuSO_4_·5H_2_O 1 g/L, Na_2_B_4_O_7_·10H_2_O 1 g/L, NH_4_)_6_Mo_7_O_24_·4H_2_O 1 g/L) in 100 mL.
